# Apple, condom, and cocaine – body stuffing in prison: a case report

**DOI:** 10.1186/s13256-018-1572-8

**Published:** 2018-02-11

**Authors:** Benedicte Jalbert, Nguyen Toan Tran, Stephan von Düring, Pierre-Alexandre Poletti, Ian Fournier, Catherine Hafner, Celestine Dubost, Laurent Gétaz, Hans Wolff

**Affiliations:** 1 Department of General Internal Medicine, Geneva University Hospitals and Faculty of Medicine, University of Geneva, Geneva, Switzerland; 2 Division of Prison Health, Geneva University Hospitals and Faculty of Medicine, University of Geneva, Geneva, Switzerland; 3 Department of Radiology, Geneva University Hospitals and Faculty of Medicine, University of Geneva, Geneva, Switzerland; 4 Department of Visceral Surgery, Geneva University Hospitals and Faculty of Medicine, University of Geneva, Geneva, Switzerland

**Keywords:** Body stuffing, Prison, Cocaine, Condom, Apple, Gastric retention, Radiology pitfalls

## Abstract

**Background:**

Drug dealers and drug users resort to body stuffing to hastily conceal illicit drugs by ingesting their drug packets. This practice represents a medical challenge because rupture of the often insecure packaging can be toxic and even lethal. In an emergency setting, official guidelines are needed to help the medical team decide on the proper treatment. A preliminary observation period is generally accepted but its duration varies from hours to eventual packet expulsion.

**Case presentation:**

This case involves a 20-year-old white man in detention who claimed to have ingested one cocaine packet wrapped in plastic food-wrap and a condom in anticipation of an impending cell search. He reached out to medical professionals on day 4 after having unsuccessfully tried several methods to expel the drug packet, including swallowing olive oil, natural laxatives, liters of water, and 12 carved apple chunks. An initial computed tomography scan confirmed multiple packet-sized images throughout his stomach and bowel. After 24 hours of observation and normal bowel movements without expelling any packets, a subsequent scan found only one air-lined packet afloat in the gastric content. Due to the prolonged retention of the package there was an increased risk of rupture. The packet was eventually removed by laparoscopic gastrotomy.

**Conclusions:**

This case report illustrates that observation time needs to be adapted to each individual case of body stuffing. Proof of complete drug package evacuation ensures secure patient discharge. Body stuffers should be routinely asked for a detailed history, including how the drug is wrapped, and whether or not they ingested other substances to help expel the packets. The history enables the accurate interpretation of imaging. Repeated imaging can help follow the progress of packets if not all have been expelled during the observation period. Drug packets should be surgically removed in case of prolonged retention. To ensure the best possible outcomes, patients should have access to high-quality, private, and confidential medical care, which is equal to that offered to the general population. This is paramount to earning trust and collaboration from people in detention who resort to body stuffing.

## Background

Body stuffers, who are at the end of the drug trafficking chain as community drug dealers or consumers, swallow the few drug packets that they have on hand (mostly cocaine and heroin) to conceal them from law enforcement in anticipation of impending searches or arrest [1]. In contrast, body packers usually ingest a large number of mechanically manufactured compact drug packets enclosed in multiple layers of wrapping to resist rupture during long-distance drug smuggling [2].

When taken to a medical facility, an unenhanced computed tomography (CT) scan is usually preformed and is preferred to conventional X-ray for confirmation of the diagnosis [3–5]. Body stuffers are then usually observed for a few hours and released if no complications occur [6]. However, the required duration of the observation is still debated with some authors recommending discharge after 6 hours of unremarkable observation, and others recommending a conservative approach, which consists of waiting for the evacuation of the drug packets by unaided bowel movements (no medication, such as prokinetic agents or laxatives) [7, 8]. The use of oil-based laxatives is not recommended as it can cause packet rupture due to a chemical reaction with latex [9]. However, if patients do not spontaneously expel the packets after a few days, especially if these were loosely wrapped and not intended for internal concealment, they will require further medical attention due to the increased risk of rupture. This is true even if reported body stuffer fatalities are rare and the quantity of ingested drugs is usually less than that of body packers [10–12]. However, packet rupture is dangerous and can be lethal [13, 14]. Surgery is the recommended choice to safely remove retained drug packets versus endoscopy in which the manipulation of packets increases the risk of rupture [15–17].

We present this case report to shed light on some of the particular and confusing circumstances that can be found when managing body stuffers. Our report demonstrates that a 6-hour observation period can be insufficient and that CT findings must be correlated with the patient’s story in order to determine the severity of the situation.

## Case presentation

**Fig. 1:**
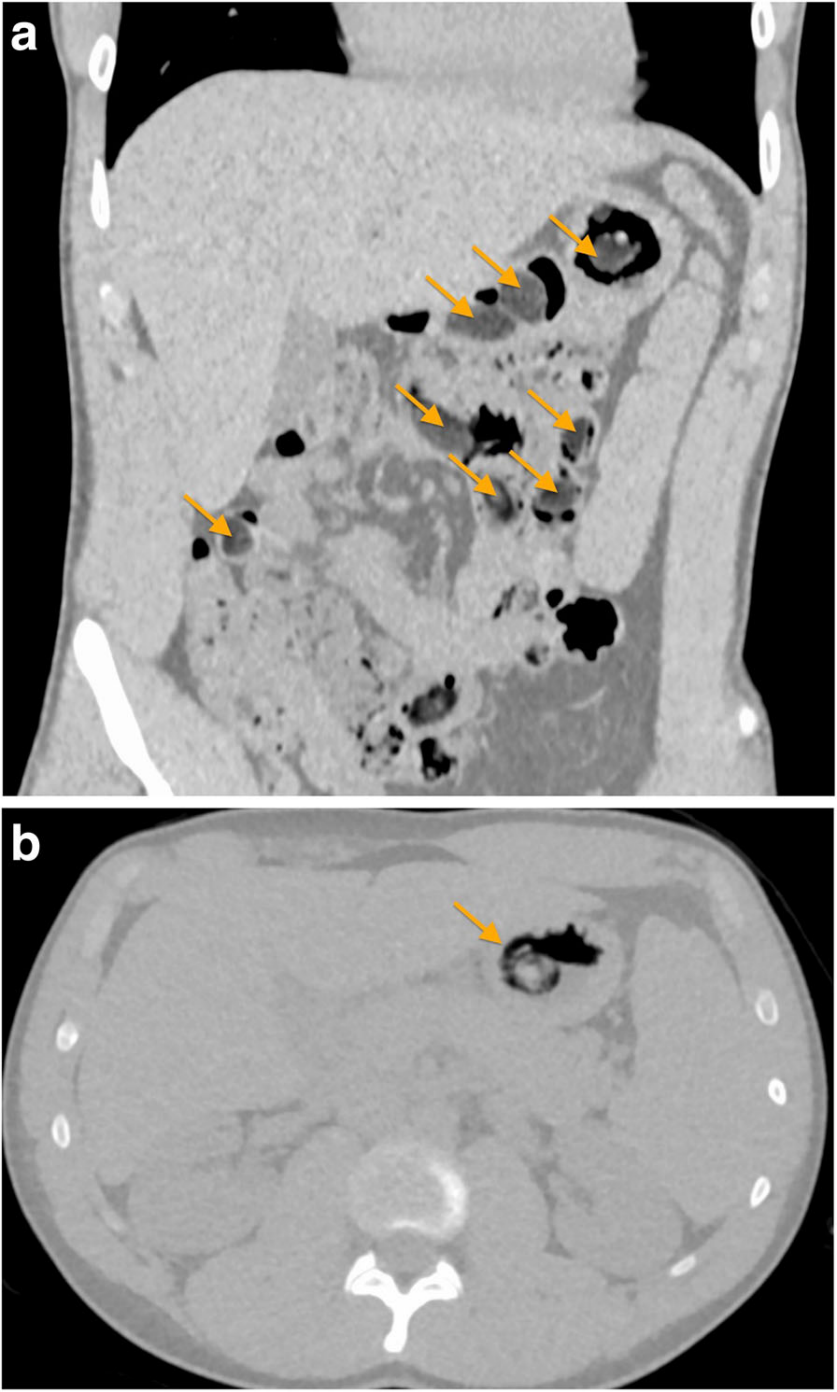
**a** Multiple intradigestive foreign bodies (*arrows*) situated in the stomach, the duodenum, and small intestine on computed tomography (coronal view). **b** Single heterogeneous foreign body (*arrow*) containing air measuring 3.1 × 3.2 × 4 cm located in the stomach on computed tomography (axial view)

This case involves a 20-year-old healthy white man incarcerated in a detention center in Geneva, Switzerland. He had no medical or mental health conditions and did not take any medication. He has two older siblings, had dropped out of school at a young age, and was living with his mother at the time of incarceration. He used to smoke tobacco and cannabis, which he stopped 2 years ago, consumed cocaine and ecstasy (methylene-dioxymethamphetamine) until 6 months before his incarceration, and drank alcohol occasionally without reporting any binge drinking. Due to a recent trauma to his left wrist, he was accompanied to our University Hospital for an X-ray. Once in Radiology, he confessed to the technician that he had ingested an illicit drug packet 4 days earlier in anticipation of an impending cell search. The drug packet was reportedly 4 to 5 cm in size, contained 6 to 8 g of cocaine, and was wrapped in a condom and plastic food-wrap. He was worried because he had not yet evacuated the packet and had been experiencing mild epigastric pain for a few hours before his x-ray appointment. He did not have any other gastrointestinal (GI) symptoms, including nausea, vomiting, diarrhea, and constipation. After the wrist X-ray he was taken to our Emergency Department (ED) for the management of the body stuffing. On admission, his vital signs were as follows: blood pressure of 131/60 mmHg, heart rate of 74 beats/minute, and temperature of 37.6 °C. He was alert, oriented, calm, and expressed no intention of self-harm. A physical examination revealed epigastric tenderness, but no abdominal rigidity, guarding, rebound tenderness, or evidence of a palpable mass. The rest of the examination was unremarkable, including a cardiopulmonary and a complete neurological examination. Laboratory findings were within normal range, including a complete blood count (hemoglobin of 16.2 g/dL, white cell count of 8.5 G/L, platelet count of 121 G/L), kidney and liver function tests, and a urine analysis. Tests for HIV and hepatitis B were negative. He returned to our Radiology Department where a low-dose abdominal CT scan was performed and revealed multiple foreign bodies of similar aspect throughout his stomach, his duodenum, and his small intestine, all of which were consistent with packets of loosely aggregated drugs (Fig. 1a). There was no sign of GI perforation or obstruction.

He was subsequently admitted to our in-patient Carceral Unit for observation. When confronted with the radiology findings of multiple foreign bodies he insisted on having swallowed only one drug packet. Further exploration revealed that he used various GI-stimulating techniques which had been recommended by his fellow inmates to accelerate the expulsion of his slow-progressing packet. These included drinking large quantities of water (more than 3 L daily), ingesting three to four tablespoons of olive oil daily, a cupful of natural fig-based laxative, and applying warm towels on his “liver.” On day 3 after the packet ingestion and as a last resort, he carved and ingested 12 bite-sized apple chunks with the hope that together they would push the packet through his GI system. Despite all these attempts, he continued to have regular bowel movements once daily without evacuating the packet. The fear of a complication compelled him to seize the opportunity of being brought to the X-ray room to reveal the circumstances of his condition to the radiology technician. Since the apple chunks could have been interpreted as images of loosely compacted drug packets, a second low-dose CT scan was performed the day after his admission (day 5 post-ingestion): the apple chunks were partially digested and the images showed the persistence in his stomach of one foreign body containing air and measuring 3.1 × 3.2 × 4 cm (Fig. 1b).

**Fig. 2:**
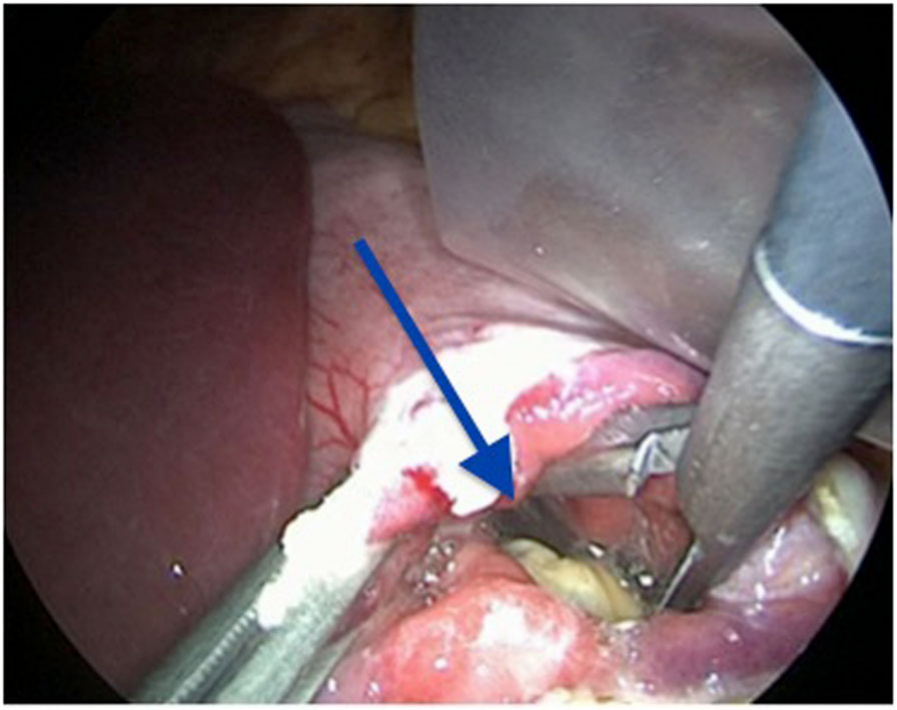
Gastrotomy by laparoscopy showing the packet floating in the stomach (*blue arrow*)

**Fig. 3:**
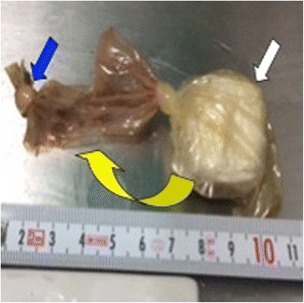
Drug packet tied in a condom with an internal knot (*white arrow*); the second layer of the condom which was folded back to form another protective layer and tied with an outer knot (*blue arrow*) was cut open and pulled to the left (*yellow arrow*)

**Fig. 4:**
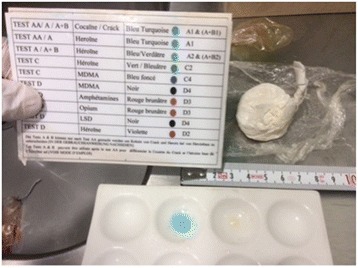
Unwrapped drug packet from a double layer of plastic food-wrap with a positive cocaine identification (*in blue*)

Since the packet had been trapped in his stomach for the past 5 days, the condom wrapping was likely to have been compromised by gastric acidity, thus increasing the risk of rupture. Therefore, a proton pump inhibitor (PPI) of esomeprazole was administered intravenously. After discussing management options with our patient, GI specialists recommended using surgery rather than gastric endoscopy in order to extract the drug packet with a minimum of risk. A laparoscopic gastrotomy confirmed a floating packet in the gastric fluid which was removed without complications (Fig. 2). Analysis of the packet after retrieval confirmed that the drug was cocaine. The packet was loosely wrapped in two different materials: a first double layer of plastic food-wrap inserted into a condom which was tied with a knot and folded back to form another layer and secured with an outer knot (Figs. 3 and 4).

He presented a transitory fever up to 38.8 °C at 48-hours post-surgery but otherwise maintained normal vital signs. He resumed oral intake shortly after surgery and continued the PPI treatment for 2 weeks after surgery to aid healing. He was discharged back to prison on day 4 post-surgery. An out-patient follow-up in the prison health service was unremarkable. He was released from detention 2 months after surgery and further follow-up was not possible.

## Discussion

This is the case of a 20-year-old man who ingested apple chunks to help with the expulsion of a cocaine packet, which was eventually removed by laparoscopic gastrotomy. It illustrates several points that are necessary to consider for the management of body stuffers.

First, contrary to the recommendations of Yamamoto *et al*., an adequate observation time in a medical setting is necessary even if body stuffers do not show signs of complication during the first 6 hours following packet ingestion [7]. Body stuffers often resort to precarious packaging for hasty concealment. Therefore, care must be organized around this point. They present an increased risk of packet rupture and unpredictable progression through the digestive system resulting in the possibility of delayed toxicity even if no signs of toxicity are observed within the first 6 hours post-ingestion. This case shows that loosely aggregated drugs within a poorly wrapped package with trapped air between the different layers may not have the sufficient density to descend and engage into the pylorus. This is compounded if the patient drinks large amounts of fluids which may facilitate normal transit but will keep the packet buoyant in the stomach. A premature discharge of our patient from the hospital could have resulted in poor outcomes and even death (the estimated weight of 6 to 8 g of cocaine, even with admixture, surpasses the minimum lethal dose of 1.2 g of pure cocaine) [10]. Until now, no official guidelines have been set to determine the correct duration of surveillance of body stuffers. Therefore, we believe that it is essential to incorporate the influence of these different technical parameters into the duration of the observation time and discuss with the patient the risks and benefits of a prolonged surveillance to determine the most appropriate course of action.

Second, localizing the number, location, and progression of packets is necessary for clinical management. The detection of internal drug packets must be performed by low-dose CT without contrast rather than by conventional X-ray as CT is superior in providing an accurate and reliable detection of different densities, from low-density to high-density packets and different densities of drug aggregation [18]. However, there can be pitfalls due to foreign bodies of similar density, especially if located in the stomach, which can mimic the images of drug packets, as was the case with the pieces of apple in our case study. The literature reports other swallowed foreign bodies that can resemble drug packets, including scybala (hardened masses of feces), grains, stones, or fruits [19]. A way to differentiate the nature of the ingesta would be to measure the Hounsfield unit on CT, which reflects density. However, density measurements are not completely reliable as the nature of the substance, such as its purity, admixture, and compression, plays a major role in imaging [18]. Therefore, body stuffers should be routinely asked whether they ingested other substances to help expel the packages and their history should help interpret the CT images.

Third, latex wrapping warrants some attention. Latex condoms can be used for packaging drugs in body stuffing and less rarely in body packing, where packages are generally manufactured mechanically to ensure robustness. It is well documented that oil-based lubricants have an adverse effect on the physical properties of latex condoms (tensile strength, break at elongation, burst pressure, and volume), which can result in their rupture [9]. Such lubricants are found in laxatives, which is the reason their use is not routinely recommended for accelerating the evacuation of drug packets. Gastric juices are extremely corrosive due to their acidity. Therefore, neutralizing the acidity with a PPI appeared to be an empirically safe way to delay the corrosion and rupture of a condom-wrapped package trapped in the stomach. However, surgical management in our case was the safest option.

Fourth, body stuffers usually do not trust authorities and are not prone to admitting their drug concealment to anybody. Therefore, they are at increased risk of complications, even more so if they are already in detention. The Geneva University Hospitals have a long-held tradition of providing health services in the detention centers in the canton of Geneva. These services are independent of the judiciary and prison system and are guided by a human rights-based approach to guarantee free access to a health care provider, equivalence of care, and patient’s consent and confidentiality [20]. The respect of these principles gives confidence to people living in detention toward health care professionals: it enables them to seek help that they trust whenever needed. In our case, the patient reached out to a radiology technician (and not to a prison staff member), and collaborated with the ED and in-patient medical team, knowing that the circumstances of his medical intervention would be known only to the medical team and would not be divulged to the penal authorities.

## Conclusions

This case report illustrates that sufficient observation time in an appropriate medical setting is essential for patients presenting with drug body stuffing. Complete drug package evacuation ensures secure patient discharge. A detailed history is fundamental in body stuffers, including how the drug is wrapped, and whether or not other substances were ingested to help expel the packets. The history must be taken into account in the careful interpretation of CT images, and imaging should be repeated if the progression of the packets is in doubt. Drug packets should be surgically removed in case of retention. To ensure the best possible outcomes, providing access to private, confidential, and high-quality medical care, which is equal to the care offered to the general population, is paramount to gain trust and collaboration from people in detention who resort to body stuffing.

## Abbreviations

CT: Computed tomography
ED: Emergency Department
GI: Gastrointestinal
PPI: Proton pump inhibitor
